# Posterior Circulation Stroke After Subclavian Artery Thrombosis Following Low-Voltage Electrical Injury in an Active Woman With Previously Unrecognized Prothrombotic Risk Factors: A Case Report

**DOI:** 10.7759/cureus.115790

**Published:** 2026-09-04

**Authors:** Logan P MacLennan, John Ashurst, Andrei Balandin

**Affiliations:** 1 Research, Midwestern University Arizona College of Osteopathic Medicine, Glendale, USA; 2 Medical Oncology, Ironwood Cancer and Research Centers, Glendale, USA

**Keywords:** case report, embolic stroke, factor v leiden, jak-2 mutation, low-voltage electrical injury, posterior circulation stroke, subclavian artery thrombosis

## Abstract

A 45-year-old active woman without previously known significant medical history presented with acute neurologic deficits (National Institutes of Health Stroke Scale (NIHSS) score 3) following a low-voltage electrical shock to her right arm three days earlier. Computed tomography angiography demonstrated a thrombus in the proximal right subclavian artery just distal to the origin of the right vertebral artery, while brain imaging revealed multifocal posterior circulation infarcts involving the left thalamus, left occipital lobe, and right cerebellar hemisphere. An extensive evaluation did not identify a cardiac source of embolism, intracardiac shunt, or convincing arterial dissection. Hypercoagulable testing identified heterozygous Factor V Leiden, and subsequent outpatient hematologic evaluation established a diagnosis of JAK2-mutated essential thrombocythemia (ET), revealing previously unrecognized prothrombotic risk factors. The patient was treated initially with intravenous unfractionated heparin and subsequently transitioned to rivaroxaban, with complete radiographic resolution of the subclavian thrombus and neurological recovery to NIHSS 0 and modified Rankin Scale (mRS) 0 at discharge. Following the diagnosis of ET, aspirin therapy was initiated, and she remained on rivaroxaban at the time of manuscript preparation. Although causality cannot be established, the temporal relationship and anatomic distribution raise the possibility that electrical injury acted as a vascular trigger in a patient with an underlying predisposition to thrombosis. This case emphasizes the importance of vascular imaging and evaluation for competing prothrombotic conditions when neurologic deficits develop following electrical injury.

## Introduction

Electrical injuries, particularly low-voltage exposures (<1,000 V), are most commonly associated with cutaneous burns and myonecrosis, while vascular complications are considered rare [1,2]. Nonetheless, thrombosis, vasospasm, and arterial dissection have been reported and are thought to arise from endothelial injury or smooth muscle necrosis induced by electrical current [1,3]. This endothelial injury and necrotic tissue may then promote thrombogenesis with subsequent embolization and infarction of the thrombus in downstream vascular beds, including strokes involving the posterior cerebral artery (PCA) [4,5]. These account for 5%-10% of ischemic strokes and commonly present with visual field deficits, cognitive or memory impairment, sensory disturbances, and higher cortical visual dysfunction [6]. Here, we describe a unique case of a young healthy woman who developed multifocal posterior circulation infarcts associated with a subclavian artery thrombus following low-voltage electrical exposure.

## Case presentation

A 45-year-old avid marathon runner with no history of prior vascular or neurologic interventions presented with confusion, right upper extremity paresthesia, and visual disturbances. Three days prior to symptom onset, she sustained a low-voltage electrical shock to her right arm while plugging in an appliance, which was followed by transient right arm numbness, fatigue, cognitive slowing, and intermittent confusion. She first presented to an outside facility in early August, where records were unavailable and diagnostic evaluation was limited by unavailable magnetic resonance imaging (MRI). This contributed to a delay in definitive vascular imaging and led to later transfer to another hospital due to worsening right-sided numbness and visual changes, at which point records became available. The patient denied a personal history of thromboembolism. Family history was notable for a paternal grandmother with stroke in her 70s and a paternal aunt with pregnancy-associated deep vein thrombosis (DVT), with no other known clotting disorders or premature cerebrovascular disease. She was not taking prescription medications, including hormonal therapy or oral contraceptives, had no history of smoking, and she denied recent infection or prolonged immobilization.

On presentation, her vital signs were stable with a blood pressure of 116/92 mmHg, pulse of 58 bpm, respiratory rate of 18 breaths/min, and peripheral oxygen saturation of 98% on room air. On neurological examination, the patient was alert and fully oriented, with intact attention and the ability to recall details of her clinical history, although subjective forgetfulness was noted in her history. Language was fluent without aphasia, and speech was clear without dysarthria, aside from a chronic mild lisp. Cranial nerve examination demonstrated a right homonymous hemianopia with pupils equal, round, and reactive to light bilaterally; extraocular movements were intact without nystagmus; facial sensation and strength were normal and symmetric; hearing was intact; the palate elevated symmetrically; shoulder shrug was full; and the tongue was midline without atrophy or fasciculations. Motor examination revealed full strength (5/5) in all muscle groups of the upper and lower extremities, with normal tone and no pronator drift. Sensory testing showed slightly decreased light-touch sensation over the right upper and lower extremities, most pronounced in a C5 distribution in the right upper extremity. Deep tendon reflexes were brisk but symmetric at the biceps, triceps, brachioradialis, patellar, and Achilles tendons. Bilateral Hoffmann signs and crossed adductor responses were present, with flexor plantar responses. Coordination was intact with normal finger-to-nose and heel-to-shin testing bilaterally, and gait was not reported as abnormal. The overall National Institutes of Health Stroke Scale (NIHSS) score was 3, consisting of 2 points for the right homonymous hemianopia and 1 point for the mild right-sided sensory deficit [7].

**Figure 1:**
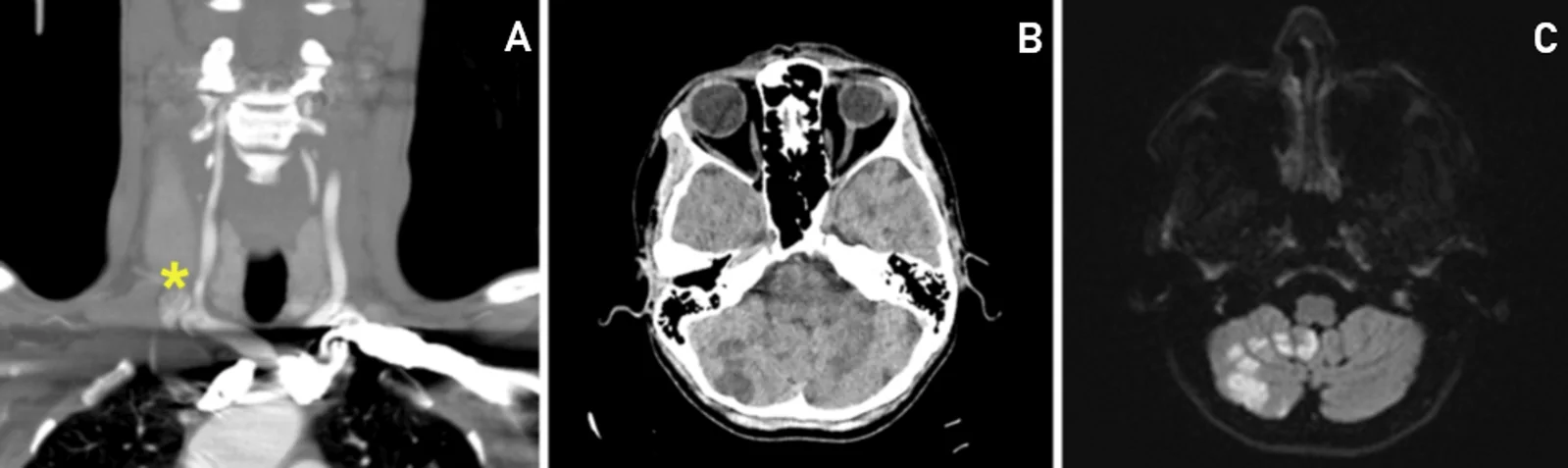
Computed tomography (CT) and magnetic resonance (MR) imaging demonstrate thrombus formation and downstream damage in susceptible vascular territories. (A) CT angiography (CTA) of the head and neck shows thrombus (*) identified within the right subclavian artery just distal to the takeoff of the right vertebral artery. (B) Head CT without intravenous (IV) contrast shows multifocal hypodensities within the right inferior cerebellar hemisphere involving the posterior inferior cerebellar artery distribution with no evidence of hemorrhagic conversion. (C) Brain MR shows multifocal increased diffusion-weighted imaging (DWI) signal with associated mildly decreased apparent diffusion coefficient (ADC) and T2 fluid-attenuated inversion recovery (FLAIR) hyperintensity throughout the right cerebellar hemisphere. The multifocal nature and distribution of infarctions suggest an embolic source.

Computed tomography (CT) and CT angiography (CTA) of the head and neck demonstrated multifocal acute infarcts involving the left thalamus, left occipital lobe, and right cerebellum, along with a thrombus in the proximal right subclavian artery just distal to the vertebral artery origin. MRI of the brain confirmed multiple posterior circulation infarcts. This imaging can be viewed in Figure 1. Follow-up magnetic resonance angiography (MRA) of the neck showed a decrease in thrombus size with mild irregularity of the V3 segment of the vertebral artery, considered to represent either flow artifact or a small arterial dissection. However, CTA and MRA demonstrated no convincing intimal flap, mural hematoma, or characteristic vessel narrowing to establish arterial dissection. The differential diagnosis included cervical arterial dissection, cardioembolism, paradoxical embolism, vasculitis, and an underlying hypercoagulable disorder. Transthoracic and transesophageal echocardiography revealed no intracardiac thrombus, shunt, or structural abnormality with a negative bubble study, and telemetry showed no arrhythmia, making a cardioembolic source unlikely. CT of the chest showed no evidence of pulmonary embolism.

Admission and diagnostic laboratory findings are summarized in Table 1. Initial evaluation demonstrated mild hemoconcentration (hemoglobin 16.2 g/dL), thrombocytosis (515 × 10³/µL), and mild leukocytosis (11.0 × 10³/µL), while troponin, urinalysis, urine drug screen, ethanol level, and comprehensive metabolic panel were otherwise unremarkable. Hypercoagulable evaluation identified a heterozygous Factor V Leiden mutation, whereas autoimmune and inflammatory testing, including antinuclear antibodies, antineutrophil cytoplasmic antibodies, and antiphospholipid antibodies, was negative. The thrombocytosis identified during the acute presentation subsequently prompted further outpatient hematologic evaluation. In the months following hospitalization, the patient was diagnosed with JAK2-mutated essential thrombocythemia (ET), establishing an additional and clinically significant underlying prothrombotic condition.

**Table 1:** Admission and diagnostic laboratory findings. *The comprehensive metabolic panel was within normal limits except for a transient potassium elevation (5.6 mmol/L).

Laboratory test	Patient value	Reference range	Units
Hemoglobin	16.2	12.0-15.5	g/dL
Platelet count	515	150-450	×10³/µL
White blood cell count	11.0	4.0-10.0	×10³/µL
Comprehensive metabolic panel	Within normal limits*	-	-
Troponin T (baseline)	<6	<14	ng/L
Troponin T (2 hours)	<6	<14	ng/L
Urinalysis	Negative	Negative	-
Urine drug screen	Negative	Negative	-
Ethanol level	Negative	Negative	mg/dL
Factor V Leiden mutation	Heterozygous mutation detected	Negative	Qualitative
Antinuclear antibodies (ANA)	Negative	Negative	Qualitative
Antineutrophil cytoplasmic antibodies (ANCA)	Negative	Negative	Qualitative
Antiphospholipid antibody panel	Negative	Negative	Qualitative

The patient was initially treated with weight-based intravenous unfractionated heparin titrated to a therapeutic activated partial thromboplastin time (aPTT) level. She participated in physical, occupational, and speech therapy and demonstrated marked clinical improvement over the course of one week. On hospital day 6, she was transitioned to rivaroxaban 15 mg twice daily for 21 days followed by 20 mg once daily. At discharge on hospital day 7, she had achieved complete neurological recovery with an NIHSS score of 0 and a modified Rankin Scale score of 0. Follow-up vascular imaging demonstrated complete resolution of the subclavian artery thrombus.

Following outpatient diagnosis of JAK2-mutated ET, aspirin therapy was initiated. She remained on rivaroxaban at the time of manuscript preparation. She tolerated anticoagulation without documented bleeding complications and reported adherence to therapy.

## Discussion

Electrical injuries can produce vascular damage through several mechanisms, including direct endothelial injury, thermal damage to the vascular wall, and electrically induced vasospasm. These processes may disrupt normal blood flow and create conditions favorable for thrombosis [1,3]. Severe vascular injury is more commonly described following high-voltage exposure, in which greater energy transfer can result in substantial tissue and vascular damage [6]. Nevertheless, clinically significant vascular complications have also been reported following low-voltage electrical injury [1].

Ischemic stroke is an uncommon but reported complication of low-voltage electrical exposure. Huan-Jui et al. described ischemic stroke associated with segmental cerebral arterial narrowing after low-voltage injury, with vasospasm proposed as a potential mechanism [1]. Kokatnur and Rudrappa similarly reported acute stroke following electrocution [2]. Chen et al. described brainstem ischemic stroke after a short-duration 110 V exposure in a patient with diabetes and decreased protein C activity, suggesting that underlying vascular/hemostatic abnormalities may increase susceptibility to thrombosis following electrical injury [8]. Peripheral arterial thrombosis has also been reported after apparently limited electrical exposure; Bongard and Fagrell described delayed arterial thrombosis following a 220 V injury [9].

In the present case, the proximal right subclavian thrombus provides a plausible vascular relationship to the patient's posterior circulation infarctions. The thrombus was located immediately distal to the origin of the right vertebral artery. Although direct embolization from this location cannot be established, local hemodynamic disturbance, transient thrombus propagation, or retrograde embolization represent potential mechanisms by which thrombotic material could have entered the vertebrobasilar circulation. The multifocal distribution of cerebral infarction is also compatible with an embolic process.

An important competing explanation, however, is the patient's subsequent diagnosis of JAK2-mutated ET. ET is a chronic myeloproliferative neoplasm associated with an increased risk of both arterial and venous thrombosis, and JAK2 mutation is an important predictor of thrombotic risk. Contemporary risk stratification incorporates age, previous thrombosis, and JAK2 mutation status, with prior thrombosis placing patients in a high-risk category regardless of age [10]. In retrospect, the patient's platelet count of 515 × 10³/µL at presentation was notable, and persistent thrombocytosis ultimately prompted hematologic evaluation and the diagnosis of JAK2-mutated ET.

The relationship between ET and the vascular findings in this case is particularly important because subclavian artery thrombosis with embolic complications has previously been reported in ET. Kumar and Kiley described in situ subclavian artery thrombosis with multiple embolic events in a patient ultimately diagnosed with ET [11]. Therefore, the temporal association between electrical exposure and thrombosis in our patient cannot establish electrical injury as the sole cause.

Instead, the event may be best understood as multifactorial. Electrical injury provides a plausible mechanism for acute endothelial injury and abnormal vascular flow through vasospasm [1,3,9]. Additionally, JAK2-mutated ET provides a substantial underlying thrombotic predisposition [10,11]. A similar interaction between low-voltage electrical exposure and pre-existing coagulation abnormalities has previously been proposed [8]. The temporal relationship remains notable: the patient had no known history of thrombosis, experienced electrical exposure involving the ipsilateral arm, developed neurologic symptoms shortly thereafter, and was subsequently found to have a proximal subclavian artery thrombus on the same side. However, given the independent thrombotic risk associated with ET, electrical exposure should be considered a possible precipitating factor rather than an established sole cause [10,11].

The patient was initially treated with intravenous unfractionated heparin and subsequently transitioned to rivaroxaban, with complete radiographic resolution of the subclavian thrombus and full neurologic recovery. Following the diagnosis of ET, she was also started on aspirin. Prevention of recurrent thrombosis is a primary therapeutic goal in ET, with low-dose aspirin used for thrombosis prevention and cytoreductive therapy generally recommended for high-risk disease [10]. Long-term antithrombotic management in this unusual setting should be individualized in conjunction with hematology because standard venous thromboembolism recommendations cannot be directly extrapolated to arterial subclavian thrombosis in the setting of a persistent myeloproliferative neoplasm [10,12].

This case has several limitations. As a single case report, it cannot establish causality between electrical exposure and arterial thrombosis. The precise voltage and duration of electrical contact were not available, limiting characterization of the exposure. Additionally, no histopathologic evaluation of the thrombus or vascular wall was performed to demonstrate direct electrical vascular injury. Most importantly, JAK2-mutated ET represents a strong competing explanation for the arterial thrombosis, particularly given the previously reported association between ET and subclavian thrombosis [10,11]. These limitations support cautious interpretation of the electrical injury as a potential precipitating event rather than the definitive cause of thrombosis.

Despite these limitations, this case highlights an uncommon vascular presentation following electrical exposure and the importance of considering underlying thrombotic susceptibility. Previous reports demonstrate that low-voltage electrical injury can be associated with ischemic stroke and arterial thrombosis [1,2,8,9]. ET independently carries substantial thrombotic risk [10,11]. In patients who develop neurologic symptoms after electrical injury, vascular complications should remain part of the differential diagnosis. Identification of a potential environmental trigger should also not preclude evaluation for an underlying prothrombotic disorder when the clinical presentation warrants further investigation.

## Conclusions

This case describes proximal subclavian artery thrombosis associated with multifocal posterior circulation ischemic stroke shortly after a low-voltage electrical injury in a 45-year-old woman with no previously recognized thrombotic disorder. Subsequent evaluation revealed heterozygous Factor V Leiden and JAK2-mutated ET, substantially altering the interpretation of the event. Although the temporal and anatomic relationship raises the possibility that electrical exposure contributed to local vascular injury or altered blood flow, the available evidence cannot establish electrical injury as the sole cause of thrombosis. Instead, the event may represent the convergence of an acute vascular trigger with previously unrecognized thrombotic susceptibility. Prior reports of low-voltage electrical injury associated with stroke in patients with coagulation abnormalities support the biologic plausibility of this interaction, while the independent association between ET and subclavian arterial thrombosis underscores the importance of cautious causal attribution.

Clinicians evaluating neurologic symptoms following electrical injury should remain aware of uncommon vascular complications and consider vascular imaging when the clinical presentation suggests cerebral ischemia. Identification of an apparent environmental trigger should not preclude evaluation for an underlying thrombotic disorder, particularly when arterial thrombosis occurs in a younger patient without conventional vascular risk factors.
